# Post-exercise heart rate recovery and its speed are associated with resting-reactivity cardiovagal modulation in healthy women

**DOI:** 10.1038/s41598-024-51842-w

**Published:** 2024-03-06

**Authors:** Richard Xavier da Fonseca, Carlos Janssen Gomes da Cruz, Edgard de Melo Keene Von Koening Soares, Giliard Lago Garcia, Luiz Guilherme Grossi Porto, Guilherme Eckhardt Molina

**Affiliations:** 1 Universidade de Brasília, Faculdade de Educação Física, Programa de Pós-graduação em Educação Física, Laboratório de Fisiologia do Exercício, Brasília, DF 70910-900 Brazil; 2 Grupo de Estudo em Fisiologia e Epidemiologia do Exercício e da Atividade Física (GEAFS), Universidade de Brasília, Brasília, DF Brazil; 3 University Center Euro Americano, Brasília, DF Brazil; 4 Skidemore College, Saratoga Springs, NY USA; 5 University Center Institute of Higher Education of Brasília - IESB, Brasília, DF Brazil

**Keywords:** Physiology, Cardiology

## Abstract

The present study sought to expand upon prior investigations of the relationship between post-exercise heart rate recovery (HRR) and cardiovagal resting-reactivity modulation. HRR from 1st to 5th min after maximal exercise test was correlated with a cardiovagal index of heart rate variability (SD1) at resting (supine and orthostatic positions) and its reactivity after the orthostatic stress test in 34 healthy women. Statistical analysis employed non-parametric tests with a *p*-value set at 5%. HRR, ∆%HRR, and coefficient of HRR (CHRR) at the 3rd and 5th min correlated with SD1 and SD1_n_ (normalized units) in the supine position (r_s_ = 0.36 to 0.47; *p* =  < 0.01). From the 1st to 5th min, HRR, ∆%HRR, and CHRR correlated with SD1 and SD1_n_ in the orthostatic position (r_s_ = 0.29 to 0.47; *p* =  ≤ 0.01 to 0.05), except for HRR at 5th min with SD1_n_ (*p* = 0.06). Following the orthostatic stress test, HRR at 3rd and HRR, %∆HRR at 5th min correlated with ∆absSD1 (r_s_ = 0.28 to 0.35; *p* = 0.02 to 0.05). All HRR measurements at 1st min correlated with ∆absSD1_n_ (r_s_ = 0.32 to 0.38; *p* = 0.01 to 0.03), and the CHRR at 1st min correlated with ∆%SD1(r_s_ = 0.37; *p* = 0.01). After the sample was divided into high and low cardiovagal modulation subgroups, the subgroup with high modulation at rest (supine and orthostatic) and higher cardiovagal reactivity (reduction) showed faster HRR (*p* =  ≤ 0.01 to 0.05; *ES*:0.37 to 0.50). HRR throughout the 1st to 5th min positively correlates with cardiovagal modulation in the orthostatic position, and the 3rd and 5th min positively correlate with cardiovagal modulation in both postures at rest. Faster HRR following the maximal exercise test is associated with high resting-reactivity cardiovagal modulation in healthy women.

## Introduction

Post-exercise heart rate (HR) recovery (HRR) after maximal incremental exercise testing is an independent and powerful predictor of cardiovascular morbidity and mortality risk in individuals with distinctive clinical conditions^1,2^.

Immediately following the exercise testing, the short-term HRR is associated with rapid cardiovagal reactivation and progressive sympathetic deactivation^3,4^. Thus, it is reasonable to expect that these autonomic adjustments rely on resting cardiac autonomic condition and that adaptative heart-rate response to exercise-induced stress displays this cardiac autonomic condition.

The analysis of spontaneous heart rate variability (HRV) based on the oscillation in the intervals between consecutive heartbeats (R-R interval) is a suitable and reliable method for assessing the relative strength of cardiovagal and sympathovagal balance activities in different functional conditions (e.i, at rest supine and orthostatic positions) on the sinus node^5^. The Poincaré index SD1 is a reliable non-linear measure of HRV that can indirectly infer the cardiovagal influence on the sinus node^6^. This measurement does not require linearity and stationary of the R-R intervals series^7^, which can be used in different functional conditions and can be considered an alternative to linear time–frequency domains of HRV^8,9^. Another sensitive measurement to assess cardiac autonomic activity is the HRV response to gravitational stress imposed by active postural change (orthostatic stress test)^10^. This maneuver shows the shift (reactivity) in cardiac autonomic balance by reducing cardiovagal activity and enhancing sympathetic activity in variable degrees according to the resting activity condition^10,11^. Both HRV analyses can bring essential and complementary information related to the individual's cardiac autonomic control^12,13^.

Even though HRR and HRV measures are independent predictors of risk for cardiovascular morbimortality^1,5^, several studies have examined their relationship by seeming to be a promising area of clinical-functional evaluation^2,14^. However, inconclusive results have been found^14–20^. The population, measurement techniques, and exercise/recovery protocols may explain divergent findings on the relationship between HRR and resting HRV.

Apart from these confounding methodological factors, the majority of studies have examined this relationship only in samples composed of men^17,18,20,21^ or composed of men and women^16,22–24^. So, considering prior evidence that cardiac autonomic function obtained in men and women cannot be treated equally^25^, more investigations are needed to study the relationship between HRR and HRV at rest exclusively in women due to previously established sex differences in cardiac autonomic function at rest^25^ and its phasic reaction to stress^26^, as there is a lack of information on this topic.

To our knowledge, only one study has examined the association between HRR and HRV at resting in the supine positions in women^14^. However, incomplete results may arise because of the discrepancy in evaluating the association between HRR and resting cardiac autonomic modulation in different postural positions (e.i., orthostatic position)^27^. HRV modulation differs between supine and orthostatic positions, and the exercise stress is often performed in the latter, with HRR being measured in this position^28,29^. Yet, no study has evaluated the association between HRR, a dynamic measurement, with a dynamic measure of HRV (e.i., HRV reactivity following the orthostatic stress test at rest).

Therefore, considering that HRR is an adaptative dynamic phenomenon dependent on cardiovagal reactivation, it is essential to evaluate the association between both functional phenomena considering different resting cardiovagal modulations (supine and orthostatic positions) and its reactivity after the orthostatic stress test. Thus, we hypothesize that a greater HRR is associated with high cardiovagal resting-reactivity assessed by HRV at rest in healthy women.

Therefore, the present study analyzed the relationship between 5 min of HRR following maximal treadmill exercise stress test and the resting-reactivity cardiovagal modulation assessed by HRV in healthy women.

## Methods

### Study group and protocol

This analytical cross-sectional study was carried out with thirty-four women (n = 34) with a median (quartiles) age of 22.2 (20–24) years old and a body mass index of 22.8 (21.3–23.7 kg/m^2^). Participants were eligible for inclusion if they were women, healthy (no known disease), using oral contraceptives, non-athletes, and between 20 and 40 years old. The use of oral contraceptives was adopted since it is one of the most prevalent methods for family planning in women of reproductive age^30^. Exclusion criteria were non-sinus rhythm, smoking, and under any medication. They were previously instructed to abstain from stimulants and alcoholic beverages and cease any physical activity for at least 24 h before the evaluations.

All participants were informed about the risks and benefits of the investigation before beginning the test. Participants provided written informed consent, and the Institutional Ethical Committee on Human Research of the University of Brasília, Faculty of Medicine approved (PN:2.284.202) this study in compliance with the National Research Ethics System Guidelines and Declaration of Helsinki.

Initially, we collected information on lifestyle habits, conducted a clinical examination, and collected anthropometrical (body mass and height) and basic physiological data (HR, blood pressure, and respiratory rate). A 12-lead electrocardiograph recording (WinCardio v.5.0—Micromed®—Brazil) followed this examination was performed in the supine position in a quiet physiology laboratory room between 02:00 and 06:00 p.m., at ambient temperature (21–24 °C) and relative humidity of 50 to 60%. After 15 min of rest in the supine position, a valid 5-min *R-R* interval series was recorded. Subsequently, the subjects were asked to actively adopt the orthostatic posture at the bedside. After two minutes in this position, an additional 5-min *R-R* interval series was recorded following the methodology used as a routine in our laboratory to record the *R-R* interval series^31,32^. Before the second *R-R* interval recording, blood pressure was measured by the auscultatory method using a mercury sphygmomanometer and stethoscope to check the absence of postural hypotension in this position. Participants actively performed the orthostatic stress test without support, following the pre-protocol instruction to achieve the new body position in no more than 10 s^26^.

The maximum treadmill-graded exercise test was applied immediately after the *R-R* interval series recordings, and following the exercise test, all participants proceeded with the post-exercise active cool-down^33^. The participants performed all procedures up to seven days after the menstrual period, corresponding to the follicular phase^34^.

### Heart rate variability analysis

The *R-R* interval series were recorded using a valid and reliable heart rate monitor Polar® (model RS800CX), with a sampling rate of 1000 Hz^35^. Subsequently, each series was transferred to a microcomputer for offline data processing and analysis of *R-R* intervals variability, utilizing the Kubios HRV software (version 3.3.1, Kuopio, Finland).

Before the HRV analyses, each series of *R-R* intervals was visually verified, beat-to-beat, for sinus rhythm validation and non-sinus rhythm identification, artifacts, ectopic beats, and signals reproducibility. The automated artifact/spurious beats identification and removal were performed using the threshold method, which consists of select *R-R* intervals that are largest or smaller than 0.45 s (very low), 0.35 (low), 0.25 s (medium), 0.15 s (strong), or 0.05 s (very strong) compared to average *R-R* intervals^36^. We used the medium threshold that only removed the visually observed ectopic points as long as the tracing did not lose the physiological pattern and the removal did not exceed 1% of the recording^36^.

Qualified *R-R* interval series were highly steady and stationary as estimated by percent differences of the means and the standard deviations between three divided series segments. The variability of *R-R* interval series was analyzed in Poincare's Plot analysis, a geometric method that provides a graphical representation of the correlation of successive *R-R* intervals. Its interpretation allows dynamic analysis of the variability of the *R-R* interval series^7^. The graphical representation is in the form of an ellipse that one quantitative indicator can define: SD1, considered an instantaneous index of the variability of the R-R series representing the cardiovagal modulation^6^. In addition, to avoid any physiological (i.e., HR) or mathematical bias from HRV calculation^37,38^, the SD1 was also calculated in normalized units (SD1_n_) obtained by dividing the absolute value by the corresponding average R-R interval series and multiplying by 1000^38^.

### Cardio-pulmonary exercise test and post-exercise heart rate recovery

The cardiopulmonary exercise test consisted of a graded treadmill exercise test according to the individualized ramp protocol^39^, which started at a speed of 3 km/h and a 2.5% grade; the grade remained constant over the test, and the speed was increased gradually until volitional fatigue set in.

All tests were performed between 8 and 12 min on a conventional treadmill (ATL, Imbrasport, Brazil), and maximal oxygen uptake (VO_2_max) was obtained as described by^39^ and measured by pulmonary gas exchange using the Ergoespirometer–Cortex Metalyzer 3B (Biophysik, Leipzing, Germany).

Following the exercise test, the treadmill was set at a walking speed of 2.4 km/h, and the grade was maintained 2.5%^33^. Participants realized a post-exercise active cool-down for 5 min. This recovery protocol was adopted considering its technical feasibility, reproducibility, and frequent application in the clinical setting.

During the active recovery, the absolute and relative values of HRR were accessed by the HR reduction from HR peak to HR at 1st, 3rd, and 5th minutes throughout the recovery phase^33^.

The coefficient of relative HRR (CHRR) was applied to normalize the HRR for individual differences in the orthostatic HR immediately before the exercise (orthostatic HR) and maximal HR attained during the exercise test (HRpeak)^17^. This analysis depicts how much the HR recovered each minute after exercise towards the expected total recovery (HRpeak minus orthostatic HR). So, this coefficient is the relation of absolute values of HRR at each minute of post-exercise by total HR increment from orthostatic HR to the HRpeak (chronotropic reserve), which is represented by the equation CHRR = (HHR at 1st, 3rd, 5th min/HRpeak—orthostatic HR) × 100^17^.

### Statistical analysis

Most variables were considered to have a non-normal distribution by the Shapiro–Wilk test. Thus, we performed a non-parametric analysis, and variables were reported as median and quartile.

The Wilcoxon signed-rank test was used to compare the SD1 index at rest supine and orthostatic positions, and following the orthostatic test expressed as absolute (∆abs) and percent differences (Δ%), adopting the supine values as the reference (reactivity). The correlation was accessed using Spearman's Coefficient using the Bootstrap method to calculate bias-corrected and accelerated (BCa) 95% confidence intervals with one thousand samples to derive a robust estimator of the correlation coefficient analyses.

We also employed Spearman's correlation to assess the influence of confounding variables (age, BMI, arterial blood pressure, resting supine, and orthostatic HR, peak HR during exercise, and VO2 max) on HRR. The finding of a non-significant correlation resulted in no adjustment of HRR.

We additionally compared subgroups based on cardiovagal modulation (SD1) at supine and orthostatic positions using the 1st and 3rd terciles as cutoff points. The Mann- Whitney U-Test was used to compare the HRR and SD1 index (supine and orthostatic) between subgroups with low cardiovagal modulation (1st tercile) and high cardiovagal modulation (3rd tercile).

The significance level for differences and correlations was set as a two-tailed *p*-value ≤ 0.05. The effect size (*ES*) of comparative analysis was calculated using the formula: $ES=\frac{{\text{Z}}}{\surd {\text{n}}}$ where "Z" is the z-score converted from the probability value of a test statistic, and "√n" is the square root of the total sample size on which "Z" is based^40^. According to Cohen^41^, effect sizes with 0.10 were classified as small, 0.30 as medium, and ≥ 0.50 as large. The sample size required for this study was calculated by adopting an alpha error of 0.05, a beta error of 0.20 (power of 0.80), and a moderate effect size (0.50), which indicates a sample of 29 participants to detect at least a correlation coefficient of 0.5. The calculation formula is based on a two-tailed test^42^. Statistical analysis employed Prism® 6 for Windows software (GraphPad Software, Inc., USA, 2015).

## Results

The physiological variables were within the normal range in all participants. Medians (extremes) of arterial blood pressure were 113/69.5 (82–128/50–82) mmHg in the supine position and 113/74 (89–129/62–90) mmHg in the orthostatic one (*p* = 0.02). HR and respiratory rate were, respectively, 69.5 (64–77) bpm and 88 (79–94) bpm (*p* < 0.01), and 16 (11–22) rpm and 15 (11–21) rpm (*p* = 0.36). The measurement of functional variables in the pre-exercise resting period, during exercise, and post-exercise recovery are shown in Table 1.

**Table 1:** Functional variables in the different moments: pre-exercise rest, during exercise, and post-exercise recovery in 34 non-athletes’ healthy women.

	Pre-exercise rest period		Exercise period		The post-exercise active recovery period					
	Supine HR	Orthostatic HR	HRpeak	VO_2_max	1st min		3rd min		5th min	
					HHR (∆%)	CHRR (%)	HHR (∆%)	CHRR (%)	HHR (∆%)	CHRR (%)
Median	69.5	88	192	36.4	19 (9,8)	21.9	49 (26,2)	62.1	55 (29,2)	69.3
Quartiles	64 -77	79 -94	183–194	32.6–42.2	13.7–25.2 (7.3–13.3)	18.4–32.2	41–54.2 (21.8–29.1)	66.1–52.9	49.7–64.2 (26.3–33.8)	60.7–76.9
Extremes	53–89	66–101	172–202	27.8–49.8	13–34 (4.5–18.7)	10.8- 49.2	36–76 (18.2–38.8)	43.4–83.6	40–84 (20.2–42.9)	54.3–100

HR: heart rate in beats/min, VO_2_is shown in mL kg^-1^ min^-1^, HRR: heart rate recovery in absolute and relative (∆%) decrement in beats/min from maximal heart rate during exercise; CHRR: coefficient of relative HRR.

None of the possible confounding variables tested (resting supine and orthostatic HR, BMI, age, arterial blood pressure, peak HR during exercise, and VO_2max_) were significantly correlated with HRR in either the supine or orthostatic indices (r_s_ = −0.24 to 0.06; *p* = 0.08 to 0.36).

**Figure 1:**
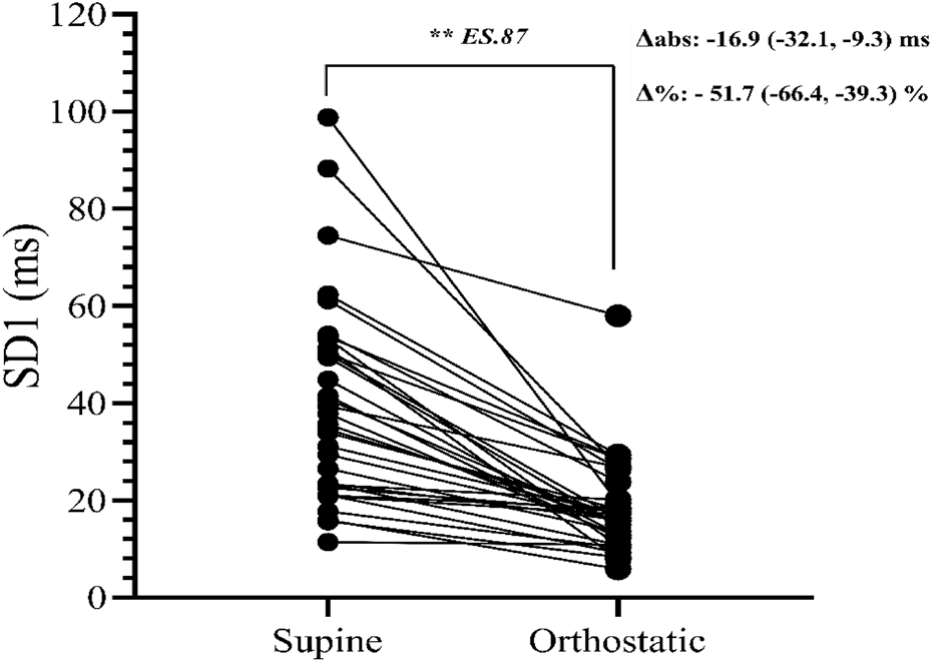
Sample values (n = 34) of resting individual cardiovagal modulation (SD1) and the mean value (Δ) of median (25th, 75th percentiles) cardiovagal reactivity values (Δabs, Δ%) of the individual values between the two body positions in non-athletes healthy women **p < 0.01. ES: Effect size.

Figure 1 shows the HRV data in the supine and orthostatic positions. Predominant cardiovagal modulation in the supine position and prominent parasympathetic withdrawal was observed in the orthostatic position (SD1: supine 36.7 (23, 51.7) ms vs. orthostatic 16.1 (11.9, 21.0) ms; *p* < 0.01; *ES*:0.87).

Table 2 shows the correlation between HRR, %∆HRR, and CHRR after exercise testing and cardiovagal modulation in the supine and orthostatic positions. HRR, %∆HRR, and CHRR at the 3rd and 5th post-exercise minutes positively correlated with SD1 in the supine position (r_s_ = 0.39 to 0.47; BCa 95% CIs = [0.08–0.70]; *p* = 0.01–< 0.01). We observed a positive correlation between HRR, %∆HRR, and CHRR from the 1st to 5th min post-exercise recovery with SD1 in the orthostatic position (r_s_ = 0.31 to 0.47; BCa 95% CIs = [0.00; 0.67]; *p* =  < 0.01 to 0.02). HRR at 3rd min and HRR, %∆HRR at 5th min were positively correlated with ∆absSD1 after the active postural change (r_s_ = 0.28 to 0.35; BCa 95% CIs = [-0.59; 0.57]; *p* = 0.02 to 0.05). On the other hand, a null correlation was found between almost all measures of HRR over the post-exercise recovery with ∆%SD1 after the active postural change, except for CHRR at 1st min, which positively correlated with ∆%SD1 after the active postural change (r_s_ = 0.37; BCa 95% CIs = [0.37; 0.65]; *p* = 0.01).

**Table 2:** Spearman’s correlation, *p*-value (in parenthesis), and bootstrap 95% confidence interval [in brackets] of the correlation of heart rate recovery (HRR), Δ% heart rate recovery (Δ%HRR), and coefficient of heart rate recovery (CHRR) at different post-exercise times with resting-reactivity cardiovagal modulation in 34 non-athletes’ healthy women.

Supine	HRR (bpm)			Δ% HRR (%)			CHRR (%)		
	1st min	3rd min	5th min	1st min	3rd min	5th min	1st min	3rd min	5th min
SD1 (ms)	0.21 (0.12) [− 0.16, 0.58]	**0.44 (< 0.01) [0.12, 0.70]**	**0.47 (< 0.01) [0.13, 0.70]**	0.21 (0.11) [− 0.15, 0.54)	**0.39 (0.01) [0.08, 0.66]**	**0.46 (0.01) [0.17, 0.69)**	0.10 (0.27) [− 0.01, 0.38]	**0.47 (0.01) [0.10, 0.69]**	**0.44 (< 0.01) [0.10, 0.69]**
Orthostatic									
SD1 (ms)	**0.43 (< 0.01) [0.12, 0.68]**	**0.38 (0.01) [0.10, 0.50]**	**0.34 (0.02) [0.03, 0.61]**	**0.47(< 0.01) [0.19, 0.67]**	**0.43 (< 0.01) [0.19, 0.67]**	**0.42 (< 0.01) [0.11, 0.64]**	**0.31 (0.03) [0.00, 0.57]**	**0.44 (< 0.01) [0.14, 0.67]**	**0.44 (< 0.01) [0.14, 0.67]**
Variation (Δ)									
∆absSD1 (ms)	− 0.21 (0.44) [− 0.33, 0.30]	**0.28 (0.05) [− 0.03, 0.55]**	**0.35 (0.02) [0.03, 0.60]**	− 0.22 (0.45) [− 0.36, 0.31]	0.21 (0.11) [− 0.14, 0.49]	**0.31 (0.04) [− 0.00, 0.57**]	− 0.24 (0.08) [− 0.51, 0.07]	− 0.15 (0.19) [− 0.44, 0.18]	− 0.10 (0.27) [− 0.42, 0.19]
∆%SD1 (%)	0.23 (0.08) [− 0.12, 0.54]	0.05 (0.37) [− 0.35, 0.25]	0.13 (0.21) [-0.41, 0.18]	0.24 (0.07) [− 0.12, 0.55]	0.16 (0.46) [− 0.31, 0.35]	0.08 (0.31) [− 0.36, 0.26]	**0.37 (0.01) [0.37, 0.65]**	0.25 (0.07) [− 0.03, 0.06]	0.13 (0.22) [− 0.40, 0.15]

Significant values are in bold.

Table 3 shows the correlation between HRR, %∆HRR, and CHRR after exercise testing and cardiovagal modulation in normalized units (SD1_n_) in the supine and orthostatic positions. HRR, %∆HRR, and CHRR at the 3rd and 5th post-exercise minutes positively correlated with SD1_n_ in the supine position (r_s_ = 0.33 to 0.46; BCa 95% CIs = [0.05; 0.70]; *p* =  < 0.01). We observed a positive correlation between HRR, %∆HRR, and CHRR from the 1st to 5th min post-exercise recovery with SD1_n_ in the orthostatic position (r_s_ = 0.28 to 0.47; BCa 95% CIs = [−0.05; 0.65]; *p* = 0.01 to ≤ 0.05) except for HRR at 5th min, which showed a statistical tendency (*p* = 0.06). HRR, %∆HRR and CHRR at 1st min were positively correlated with ∆absSD1_n_ after the active postural change (r_s_ = 0.32 to 0.38; BCa 95% CIs = [0.02; 0.64]; *p* = 0.01 to 0.03). On the other hand, a null correlation was found between all measures of HRR over the post-exercise recovery with ∆% SD1_n_ after the active postural change.

**Table 3:** Spearman’s correlation, *p*-value (in parenthesis), and bootstrap 95% confidence interval [in brackets] of the correlation of heart rate recovery (HRR), Δ% heart rate recovery (Δ%HRR), and coefficient of heart rate recovery (CHRR) at different post-exercise times with resting-reactivity cardiovagal modulation index calculated in normalized units SD1n in 34 non-athletes’ healthy women.

Supine	HRR (bpm)			Δ% HRR (%)			CHRR (%)		
	1st min	3rd min	5th min	1st min	3rd min	5th min	1st min	3rd min	5th min
SD1_n_ (ms)	0.19 (0.12) [0.15, 0.52]	**0.42 (< 0.01) [0.47, 0.70]**	**0.46 (< 0.01) [0.14, 0.72]**	0.20(0.12) [0.16, 0.53)	**0.36 (0.01) [0.07, 0.65]**	**0.43 (< 0.01) [0.08, 0.71)**	0.09 (0.47) [0.25, 0.46]	**0.33 (< 0.01) [0.05, 0.64]**	**0.42 (< 0.01) [0.07, 0.70]**
Orthostatic									
SD1_n_ (ms)	**0.37 (0.01) [0.04, 0.64]**	**0.29 (0.04) [0.08, 0.60]**	0.26 (0.06) [− 0.08, 0.57]	**0.39 (0.01) [0.06, 0.65]**	**0.30 (0.04) [0.04, 0.59]**	**0.30 (0.04) [0.03, 0.61]**	**0.28 (0.05) [− 0.05, 0.57]**	**0.32 (0.03) [− 0.01, 0.62]**	**0.32 (0.03) [− 0.01, 0.61]**
Variation (Δ)									
∆absSD1_n_ (ms)	**0.33 (0.02) [0.06, 0.58]**	0.13 (0.21) [− 0.24, 0.49]	0.13 (0.21) [− 0.24, 0.50]	**0.32 (0.03) [0.02, 0.60]**	0.10 (0.27) [− 0.26, 0.46]	0.10 (0.27) [− 0.27, 0.48]	**0.38 (0.01) [0.05, 0.64]**	0.15 (0.18) [− 0.22, 0.51]	− 0.13 (0.22) [− 0.24, 0.50]
∆%SD1_n_ (%)	0.04 (040) [− 0.34, 0.44]	0.13 (0.22) [− 0.50, 0.26]	0.13 (0.22) [− 0.50, 0.22]	− 0.04 (0.40) [− 0.36, 0.45]	0.16 (0.17) [− 0.55, 0.26]	0.17 (0.15) [− 0.55, 0.22]	− 0.06 (0.34) [0.35, 0.46]	0.14 (0.20) [− 0.54, 0.28]	0.15 (0.19) [− 0.53, 0.24]

Significant values are in Bold.

**Figure 2:**
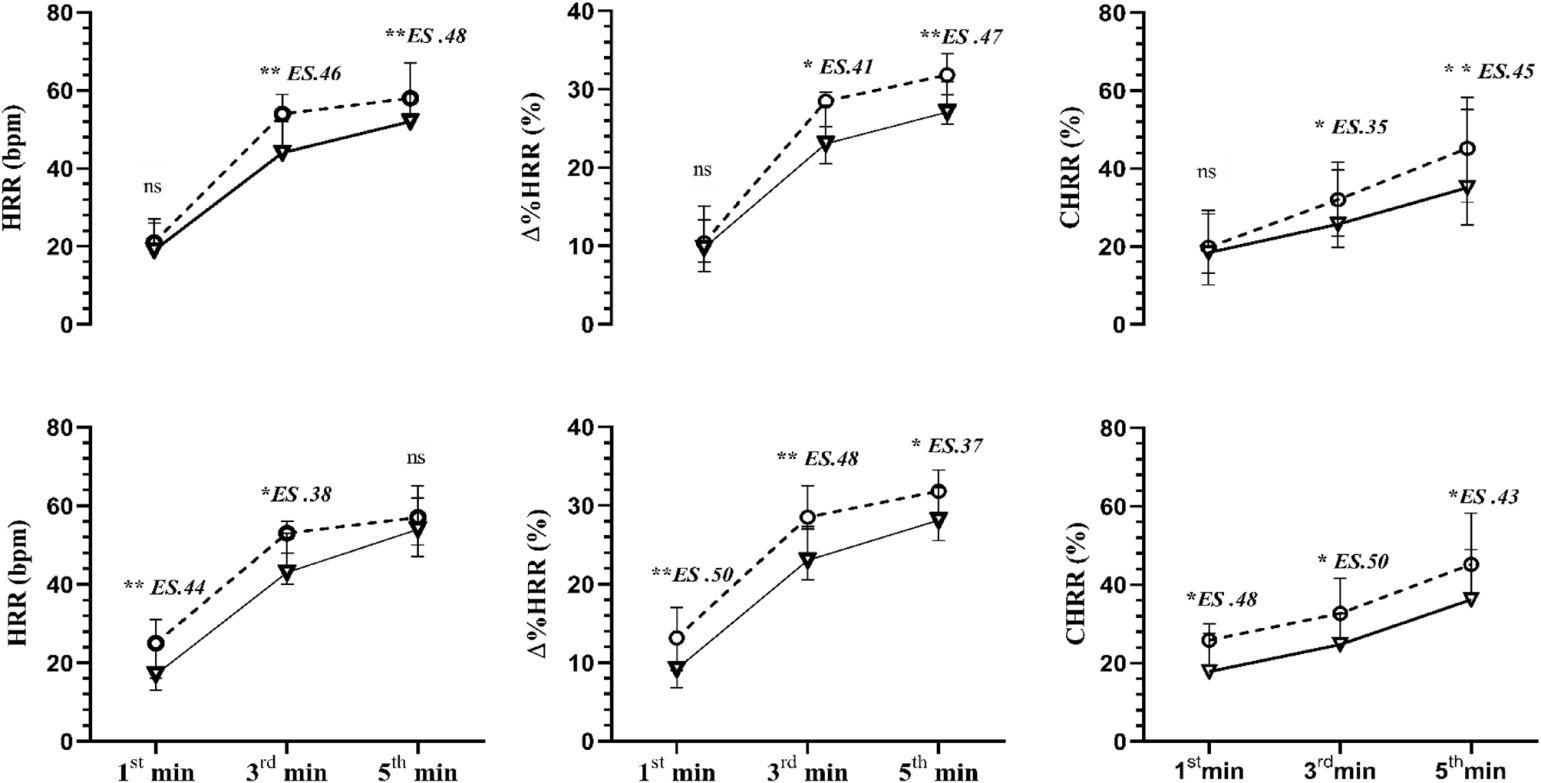
The median (25th, 75th percentiles) of the HRR, HRR% and CHRR% are shown throughout the active recovery phase in the low (**∇**) and high (**∘**) cardiovagal modulations groups. Subgroups are based on the 1st tercile (**∇**) and 3rd tercile (**∘**) of cardiovagal modulation in the supine position at rest (upper panels) and 1st tercile (**∇**) and 3rd tercile (**∘**) of cardiovagal modulation in orthostatic position at rest (lower panels). ns *P* > 0.05 * *P* ≤ 0.05 and ** *P* ≤ 0.01. Effect sizes between groups are shown above each minute. See Methods for definition of the indices.

Figure 2 shows the HRR, %∆HRR, and CHRR after exercise for two subgroups, characterized by low and high cardiovagal modulation (SD1) in supine and orthostatic positions. The subgroup that presented the highest cardiovagal modulation at rest (3rd tercile) in the supine position had a faster HRR, %∆HRR, and CHRR from 3rd to 5th min post-exercise (*p* =  ≤ 0.01 to 0.05; *ES*:0.35 to 0.48). The same pattern was observed for the subgroup that displayed high cardiovagal modulation in the orthostatic position (3rd tercile), which had a faster %∆HRR and CHRR from 1st to 5th min post-exercise (*p* =  ≤ 0.01 to 0.05; *ES*:0.37 to 0.50) and faster HRR from 1st to 3rd min (*p* =  ≤ 0.01 to 0.05; *ES*:0.38 to 0.44).

**Figure 3:**
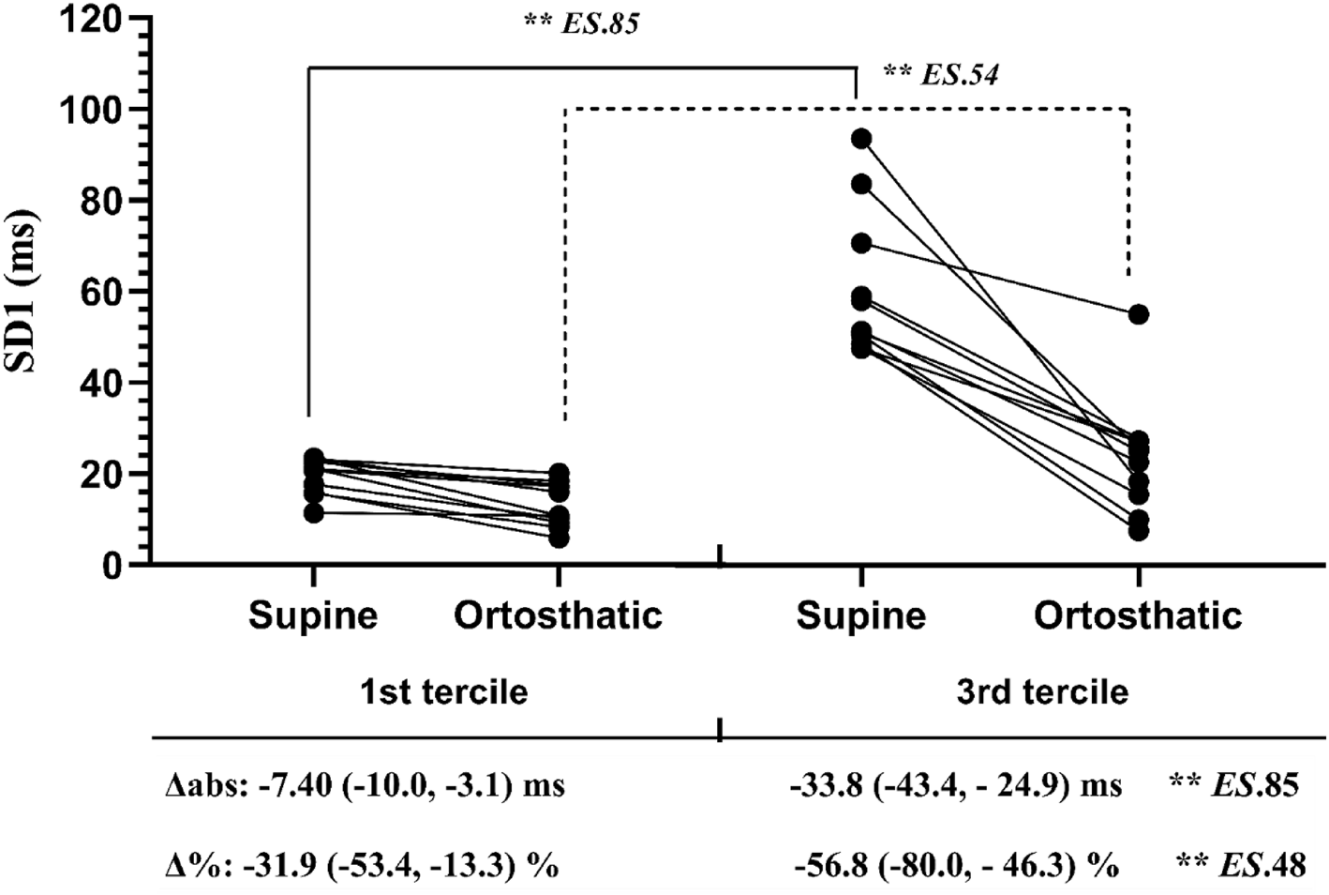
Subgroups are based on values of individual SD1 index of the non-athlete healthy women in the 1st tercile (low, n = 11) and 3rd tercile (high, n = 11) of cardiovagal modulation in the supine and orthostatic positions at rest, and the mean value (Δ) of median (25th, 75th percentiles) cardiovagal reactivity values (Δabs, Δ%) of the individual values between the two analyses, The subgroups were compared in each condition by the Mann–Whitney test. **p < 0.01, ES: Effect size.

Regarding the subgroups characterized by low and high cardiovagal modulation (SD1) at rest (supine and orthostatic positions), we observed significantly higher cardiovagal modulation in both postural positions for 3rd tercile subgroup (*p* < 0.01, ES: 0.54 to 0.85). Concerning the cardiovagal reactivity, cardiovagal variation (reduction) from the supine to the orthostatic position, we observed significantly greater absolute (*p* < 0.01; *ES*:0.85) and relative (*p* < 0.01; *ES*:0.48) reactivity (response) of SD1 index in the high cardiovagal modulation subgroup (3rd tercile) compared to low cardiovagal modulation subgroup (1st tercile) following the orthostatic stress test (Fig. 3).

## Discussion

We verified the relationship between HRR (5 min) following maximal treadmill exercise stress test with cardiovagal resting-reactivity modulation assessed by HRV (SD1 and SD1_n_) in healthy women. Our results showed new and significant findings regarding this relationship. We observed a consistent positive correlation between HRR and CHRR throughout all post-exercise time points with cardiovagal modulation in the orthostatic position. The same was observed between almost all measures of HRR throughout the post-exercise recovery with SD1_n_ in the orthostatic position, except for HHR at 5th min, which showed a statistical tendency (*p* = 0.06). These results may have potential relevance considering that these post-exercise time points, particularly the 1st minute of recovery, are usually considered a reference for clinical evaluation of HRR following a maximal treadmill exercise test^1,33^. On the other hand, no correlation of HRR at 1st min with SD1 and SD1_n_ in the supine position was observed. However, in the 3rd and 5th minutes of recovery, HRR and CHRR correlated positively with cardiovagal modulation (SD1 and SD1_n_) in the supine position.

In addition, faster HRR was observed in the subgroup with a high level of resting cardiovagal modulation (3rd tercile) in both postures (supine and orthostatic) (Fig. 2). Therefore, the nature of the relationship observed between all measures of HRR with HRV index at rest (SD1), we identified that the higher resting cardiovagal modulation, the highest were the HRR at 1st, 3rd, and 5th min and vice-versa.

Our results also showed positive correlations between HRR at the 3rd and 5th min and Δ%HRR at the 5th min post-exercise with absolute cardiovagal reactivity (ΔabsSD1), and all measures of HRR at the 1st min post-exercise recovery correlated positively with cardiovagal reactivity in normalized units (ΔabsSD1n). The same was observed for CHHR at 1st min, which correlated positively with the relative cardiovagal reactivity (Δ%SD1) after the postural change. However, no correlation of HRR throughout all post-exercise time points with relative normalized cardiovagal reactivity was observed (Table 3). Nevertheless, when we divided the sample into subgroups, we observed that the high level of resting cardiovagal modulation subgroup (3rd tercile) showed a significantly higher absolute and relative degree of reactivity (reduction) than the low cardiovagal modulation subgroup (1st tercile) after the orthostatic test (Fig. 3). Hence, these results showed that a faster HRR response following the maximal treadmill exercise test is also associated with greater absolute and relative cardiovagal reactivity (reduction) following the orthostatic test (Fig. 3).

Therefore, the implications of the relationship found between HRR and resting-reactivity cardiovagal modulation is that a better chronotropic response during the recovery phase (restoration to the initial baseline level) following the maximal treadmill exercise test is positively correlated with resting-reactivity cardiovagal modulation. In other words, our results suggested that cardiovagal reactivation during the HRR depends on the degree of cardiovagal resting-reactivity modulation before the exercise.

Despite the common physiological functional basis between HRR and cardiovagal resting-reactivity modulation, the complexity behind their relationship and influence upon each other is not fully understood. Thus, its entire picture can only be conjectured regarding the complexity of all mechanisms involved in the cardiac autonomic function and HR dynamics.

As established, HR raises during the exercise stress test in response to cardiovagal deactivation and sympathetic activation. In contrast, following the exercise, the short-term HRR adaptation occurs mainly in response to rapid cardiovagal reactivation (inhibitory activity), while slow and progressive sympathetic withdrawal occurs^4,43^. So, the coactivation of both autonomic branches occurs several minutes immediately after exercise, and consequently, hyperactivity of both autonomic branches is present for at least 1 min immediately following the exercise^3^.

Indeed, when we observed our results on the relationship between HRR and CHRR with cardiovagal modulation in the orthostatic position, and CHRR with Δ%SD1 and HRR and CHRR with ΔabsSD1_n_ after postural change, and immediately before the exercise, we could verify a significant positive correlation between HHR and CHRR at 1st min with cardiovagal resting-reactivity modulation. Therefore our results reinforce the idea that cardiovagal reactivation rapidly increases in the 1st min following exercise in the presence of the still high sympathetic activity at this time by upregulation and, or predominance by orthostasis, and the short-term HRR may be simultaneously dependent on cardiovagal reactivation and progressive sympathetic deactivation^3,28,44,45^.

One possibility is that high levels of cardiovagal modulation at rest (orthostatic position) associated with a greater absolute and relative degree of reactivity (reduction) after the postural change (orthostatic test) allows their full post-exercise reactivation resulting in high HRR. This possibility corroborates the concept that the higher levels of cardiovagal modulation at rest (orthostatic position) associated with a greater cardiovagal reactivity (reduction) from the supine position prior to an adaptative functional demand (i.e., physical exercise), the easier it is to increase the cardiovagal reactivation in response to the post-exercise excitatory stimulus or easier to increase (sympathetic inactivation) in response to an inhibitory stimulus (parasympathetic reactivation)^13,20,46^.

Our results follow a recent study published by Bechke et al.,^14^ that show a positive correlation between HRR and cardiovagal modulation, using HRV at resting only in supine position, in young, apparently healthy females. Yet, our study adds to Bechke´s study that HRR and its speeds following maximal exercise are correlated positively with cardiovagal modulation in the orthostatic position and higher levels of cardiovagal modulation (3rd tercile–subgroup) at rest, respectively. Nevertheless, our results corroborate with previous studies that have shown a positive correlation between HRR at 1–3 min^24^ and 4 min^16^ with cardiovagal modulation in the resting supine and seat positions and HRR at 3rd–4th min, but not at 5th min, with 24-h HRV analysis^15^ when cardiovagal modulation was considered in a sample composed by males and females in the same group, healthy non-athletes of broad age range.

Interestingly, in opposition to our present observations, we have recently shown that HRR, after maximal exercise testing, negatively correlates with cardiovagal modulation at rest (orthostatic position) in healthy men^17^. Afterward, in another study, we showed that faster HRR after maximal exercise testing was positively associated with the relative degree of cardiovagal reactivity (reduction) after the active postural change in healthy men^20^.

Regardless of the sex difference in cardiac autonomic function, our results show that HRR is associated with resting cardiovagal modulation in both sexes. However, the nature of correlations differs between all measures of HRR and SD1 activity at rest. Previous findings indicate that a relative dominance of cardiovagal modulation characterizes the women’s cardiac autonomic control at rest, despite greater mean HR, whereas the men's heart is characterized by relative sympathetic dominance, despite lower HR. Studies have shown that HRV is related to brain structures such as the amygdala and the ventromedial prefrontal cortex^47^. In this context, Nugent et al. have shown that amygdala activity is positively associated with parasympathetic in women but negatively associated with parasympathetic in men^48^. However, a complete explication of the potential mechanisms behind the nature of the correlation observed is beyond the present research's scope.

On the other hand, in opposition to our observations, some studies have detected no relationship between HRR within 1–2 min following maximal or sub-maximal treadmill exercise and resting HRV indices in the supine position in resistance athletes^22^ and in men and women non-athletes^16,18,19^. Twenty-four-hour HRV also did not correlate with HRR at 1–2 min in a large sample of healthy individuals of both genders^15^.

These discordant results could be explained for different reasons by (a) measurements of HRV made in only one position or distinctive positions and (b) different clinical conditions of subjects, such as age, gender, physical fitness, and non-uniformity of exercise protocols employed.

It is important to remark in our work that the position in which the HRR is measured in the orthostatic one, proper of the workload exercise protocol employed, and thus, the more appropriate correlation of HRR measures is with the resting cardiovagal modulation obtained in this position, to eliminate differences in autonomic modulation associated with specific positions^27^. Moreover, we supposed that these divergent results might be displayed by not analyzing cardiovagal reactivity to distinctive stimuli, like after an orthostatic sress test, because HRR is an adaptative dynamic phenomenon dependent on the cardiovagal reactivation^20,49^.

From a clinical point of view, our findings support the recommendation that the analysis of the relationship between HRR and cardiovagal modulation at rest should go beyond the classical resting supine position and also include an evaluation of the orthostatic position and its reactiveness. Our recommendation is supported by other authors^13^, which highlights the cardiovagal interaction patterns of the three Rs (resting, reactivity, and recovery) to assess the cardiovagal control better. Thus, systematically investigating the three Rs enables one to make predictions regarding interactions and specific cardiovagal control response patterns, which potentially would not have emerged if each time point had been investigated independently.

From a physiological perspective, our findings may encourage new studies considering the present resting approach toward developing an estimation regression model to calculate the effect of cardiovagal resting-reactivity modulation on the HRR after a maximal exercise test for a better understanding of these complex interactions. If so, it would be a low-cost and comprehensive application test to the population as a previous tool for decision-making in a clinical-functional evaluation field, without the expense of a clinical exercise test or maximal/near the maximal effort required for HRR analysis.

Another aspect that must be addressed in our study is that the correlations observed between HRR, and cardiovagal resting-reactivity were independent of anthropometrical and confounding variables, including BMI, age, resting supine and orthostatic HR, and arterial blood pressure, peak HR during exercise, and VO_2_ maximal. This observation corroborates with previous studies^10,33^, and we added that the relative homogeneous characteristics among subjects might explain why this study has not found confounding variables.

Limitations in our study include excluding older people, men of the same age group, and athletes. Also, these results cannot be precisely extrapolated to women in general because our sample was relatively homogenous, and therefore women with different clinical-functional characteristics could show distinct responses from those observed in the present study. In that sense, we prioritize internal validity instead of external to mitigate the influence of those potential confounders. Another limitation is the absence of oral contraceptive standardization. Pill composition may alter the cardiac autonomic status and, consequently, the association between post-exercise HRR and resting-reactivity cardiovagal modulation, a hypothesis that needs to be confirmed in future studies. In addition, we evaluated HRR throughout the 5 min post-exercise active cool-down phase, which should be tested for possible differences against an evaluation undertaken during a passive recovery phase and in other ergometers. The active recovery protocol was adopted considering its technical feasibility, reproducibility, and frequent application in the clinical setting.

## Conclusion

In conclusion, our results show that HRR throughout the 1st to 5th min after maximal treadmill exercise testing was positively associated with cardiovagal modulation in the orthostatic position, and the 3rd and 5th min was positively associated with cardiovagal activities in both postural positions. Faster HRR following the maximal treadmill exercise test is associated with high resting-reactivity cardiovagal modulation in healthy women.

## Data Availability

The datasets generated and/or analysed during the current study are not publicly available due to future planned analysis but are available from the corresponding author on reasonable request.
